# P-479. The Impact of the COVID-19 Pandemic on HIV Testing in Peru: An Interrupted Time Series Analysis from 2014 to 2022

**DOI:** 10.1093/ofid/ofae631.678

**Published:** 2025-01-29

**Authors:** Robinson A Yrene-Cubas, Jesus Perez-Castilla, Daniel E Reynaga-Cottle, Maria J Bringas-Queirolo, David Soriano-Moreno, Daniel Fernandez-Guzman, Jose A Gonzales-Zamora

**Affiliations:** Universidad Científica del Sur, Lima, Lima, Peru; Universidad Nacional de San Antonio Abad del Cusco, Cusco, Cusco, Peru; Universidad San Martin de Porres, LIMA, Lima, Peru; Universidad San Martín de Porres, Lima, Lima, Peru; Unidad de Investigación Clínica y Epidemiológica, Escuela de Medicina, Universidad Peruana Unión, Lima, Lima, Peru; Escuela Profesional de Medicina Humana, Universidad Nacional de San Antonio Abad del Cusco, Cusco, Cusco, Peru; Infectious Disease Division. University of Miami, Miller School of Medicine., Miami, Florida

## Abstract

**Background:**

HIV testing is crucial for early treatment and mortality reduction. In Latin America and the Caribbean, 18% of individuals are unaware of their HIV status, which leads to a late diagnosis. Furthermore, COVID-19 lockdowns had a negative impact on HIV testing rates. This study focused on comparing pre-pandemic (January 2014-February 2020) and post-lockdown (August 2020-December 2022) testing rates to support effective HIV screening and public health strategies.

**Figure 1.**
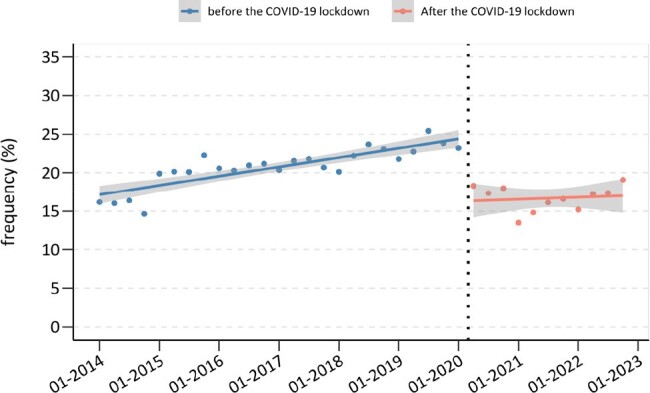
Interrupted time series analysis for the frequency of HIV testing per quarter.

**Methods:**

We performed an interrupted time series analysis with cross-sectional data from Peru's National Demographic and Health Survey (ENDES), covering 2014 to 2022. ENDES follows a complex, three-stage sampling method: proportional cluster selection, random household selection within clusters, and individual selection aged 15 or older for the health survey. A segmented regression analysis adjusted for age and gender evaluated changes in testing rates, with an ARIMA model providing counterfactual predictions against actual pandemic-period rates.

**Figure 2.**
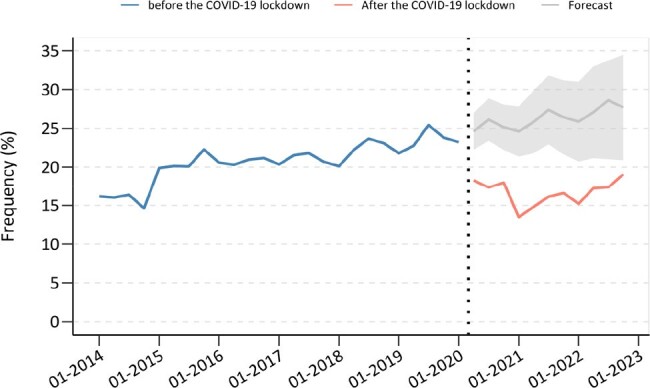
Interrupted time series analysis for HIV testing.

**Results:**

The study included 211,359 participants aged 15 to 49 years. There was a significant drop in HIV testing by 8.33% (95% CI: -10.73% to -5.93%, p< 0.001) post-lockdown. Before the lockdown, HIV testing rates trend was increasing by 0.30% per quarter (95% CI: 0.21% to 0.40%, p< 0.001); however, after the lockdown, they declined by -0.24% per quarter (95% CI: -0.56% to 0.09%, p=0.125) (Figure 1), with the most pronounced decrease among adolescents (15-17 years), the economically disadvantaged, those who lived in rural areas, and non-capital residents. The ARIMA model indicated a potential non-pandemic decline of -9.20% in testing proportions (95% CI: -13.70% to -4.80%) in comparison with the forecasted values (Figure 2).

**Conclusion:**

COVID-19 lockdown policies markedly decreased HIV testing in Peru, especially affecting high-risk groups. Although limited by potential recall bias and self-reporting, this research, focusing primarily on individuals aged 15 to 49, provides essential data for targeted interventions to restore and ensure HIV testing accessibility post-pandemic.

**Disclosures:**

**All Authors**: No reported disclosures

